# Diversity of Mitochondrial Pathology in a Mouse Model of Axonal Degeneration in Synucleinopathies

**DOI:** 10.1155/2013/817807

**Published:** 2013-03-14

**Authors:** Akio Sekigawa, Yoshiki Takamatsu, Kazunari Sekiyama, Takato Takenouchi, Shuei Sugama, Masaaki Waragai, Masayo Fujita, Makoto Hashimoto

**Affiliations:** ^1^Tokyo Metropolitan Institute of Medical Sciences, 2-1-6 Kamikitazawa, Setagaya-ku, Tokyo 156-0057, Japan; ^2^Division of Animal Sciences, National Institute of Agrobiological Sciences, 1-2 Ohwashi, Tsukuba, Ibaraki 305-8634, Japan; ^3^Nippon Medical School, 1-1-5 Sendagi, Bunkyo-ku, Tokyo 113-8602, Japan

## Abstract

There is mounting evidence for a role of mitochondrial dysfunction in the pathogenesis of **α**-synucleinopathies such as Parkinson's disease (PD) and dementia with Lewy bodies (DLB). In particular, recent studies have demonstrated that failure of mitochondrial quality control caused by loss of function of the PTEN-induced kinase 1 (PINK1, PARK6) Parkin (PARK2) pathway may be causative in some familial PD. In sporadic PD, **α**-synuclein aggregation may interfere with mitochondrial function, and this might be further exacerbated by leucine-rich repeat kinase 2 (LRRK2). The majority of these findings have been obtained in *Drosophila* and cell cultures, whereas the objective of this paper is to discuss our recent results on the axonal pathology of brains derived from transgenic mice expressing **α**-synuclein or DLB-linked P123H **β**-synuclein. In line with the current view of the pathogenesis of sporadic PD, mitochondria abnormally accumulated in **α**-synuclein/LRRK2-immunopositive axonal swellings in mice expressing **α**-synuclein. Curiously, neither mitochondria nor LRRK2 was present in the swellings of mice expressing P123H **β**-synuclein, suggesting that **α**- and **β**-synuclein might play differential roles in the mitochondrial pathology of **α**-synucleinopathies.

## 1. Introduction

The high energy consumption in neural activities in brains makes mitochondria important in neurons as regulators of energy metabolism (as ATP producers), the NAD^+^/NADH ratio, and endogenous production of reactive oxygen species (reviewed in [1]). Mitochondria are also pathologically important since abnormal mitochondria are associated with several neurological and neurodegenerative diseases, as well as with normal aging in brains [1–3]. Most importantly, mitochondrial dysfunction is a hallmark of the pathogenesis of Parkinson's disease (PD). This is because dopaminergic neurons in the substantia nigra are chronically exposed to oxidative stress caused by high levels of iron and autooxidation of dopamine. Thus, mitochondria are gradually damaged in aging, leading to enhanced oxidative stress and stimulation of neuronal dysfunction and degeneration. Mitochondria may also be acutely susceptible to environmental toxins such as drugs (e.g., 1-methyl-4-phenyl-1,2,3,4-tetrahydropyridine), herbicides, and pesticides [4]. Consistent with the idea that mitochondria are critical in neurodegeneration, complex I deficiency has been observed in postmortem PD brain and in peripheral tissues in PD patients, including platelets, lymphoblasts, muscle, and fibroblasts [5]. Furthermore, high levels of mtDNA deletions have been observed in dopaminergic neurons from the substantia nigra of postmortem human brains from elderly individuals and idiopathic PD patients [6].

Besides this circumstantial evidence, recent genetic studies of PD risk factors have unambiguously shown the central role of mitochondria in the pathogenesis of PD [7]. In early onset of familial PD, several autosomal recessive factors, such as Parkin, Pink1, and DJ-1, are clearly involved in mitochondrial pathophysiology, and loss of function of these factors might be causative for PD. On the other hand, aggregation of *α*-synuclein (*α*S) may interfere with mitochondria in sporadic PD, and mitochondrial dysfunction might be further exacerbated by dysregulation of leucine-rich repeat kinase 2 (LRRK2). However, these findings have mainly been obtained in *Drosophila* and cell cultures, and the role of mitochondrial dysfunction in the mammalian brain is still elusive. In this paper, we briefly review recent progress in genetic findings for both familial and sporadic PDs, and we give a perspective on our recent findings on the axonal pathology in transgenic mice brains expressing *α*S or DLB-linked P123H *β*-synuclein (*β*S) [8].

## 2. Role of PINK1 and Parkin in Mitochondrial Pathology and Neurodegeneration in Familial PD

Since the discovery of *α*S in a case of rare familial PD, mutations in at least 16 PD-genetic loci (PARK) have been linked to the pathogenesis of PD [1]. Among these, characterization of products for several autosomal recessive genes, including PINK-1 (PARK6), DJ-1 (PARK7), and Parkin (PARK2), has greatly accelerated research on the mitochondrial pathology of familial PD. DJ-1 may function as a redox-sensitive chaperone as well as a sensor of oxidative stress and apparently protects neurons against oxidative stress and cell death [9]. Pink-1 encodes a putative serine/threonine kinase with a mitochondrial targeting sequence [10]. Parkin has been primarily characterized as an E3 ligase in the ubiquitin proteasome system that localizes predominantly to the cytosol, but this molecule also associates with the mitochondrial outer membrane [11].

**Figure 1:**
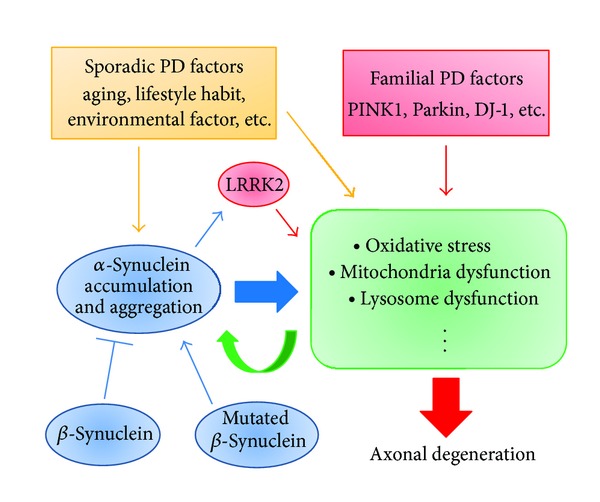
Schematics of the pathogenic mechanism of familial and sporadic *α*-synucleinopathies. Loss of function of the PINK1-Parkin pathway may be causative in the mitochondrial dysfunctions of some familial PD, while gain of functions of *α*-synuclein and LRRK2 may play a central role in the pathogenesis of sporadic PD.

Recent studies have revealed that PINK1 and Parkin may also play important roles in maintenance of mitochondrial integrity [12, 13]. PINK1 is rapidly degraded in healthy mitochondria but accumulates in membrane potential (ΔΨm) deficient mitochondria, where it recruits Parkin to ubiquitinate the damaged mitochondria, which leads to fission and processing for degradation in the lysosome; this mechanism is referred to as “mitophagy.” Thus, a prevalent view regarding the pathogenesis of some types of PD (Figure 1) is that PINK1 acts upstream of Parkin in a common pathway that appears to regulate mitochondrial quality and morphology and that dysregulation of the PINK1/Parkin pathway may result in a failure to remove damaged mitochondria, leading to enhanced oxidative stress conditions. These conditions may lead to secondary induction of aggregation of *α*S [14].

## 3. Role of **α**-Synuclein and LRRK2 in the Mitochondrial Pathology of Sporadic PD

Aggregation of *α*S may play a primary role in the pathogenesis of sporadic PD (Figure 1). In this regard, Hsu and colleagues were the first to show that accumulation of *α*S could lead to mitochondrial changes that exacerbate oxidative stress and neurodegeneration: overexpression of *α*S in hypothalamic neuronal cells resulted in formation of *α*S-immunopositive inclusion-like structures and mitochondrial changes accompanied by increased levels of free radicals, all of which were ameliorated by pretreatment with antioxidants such as Vitamin E [15]. More recently, Nakamura and colleagues showed that direct interaction of small oligomeric forms of synuclein with a membrane containing a mitochondrial lipid, cardiolipin, may be important in *α*S-expressing neurons *in vitro* and *in vivo* [16]. Notably, the effect was specific for synuclein, with more fragmentation of *α*S than of the *β*- or *γ*-isoforms, and was not accompanied by changes in the morphology of other organelles [16].

Evidence has accumulated to suggest that LRRK2 plays a crucial role in the pathology of both familial and sporadic *α*-synucleinopathies (Figure 1). LRRK2 is a large protein of 2527 amino acids that contain multiple functional domains, including leucine-rich repeats in the N-terminus, a Roc domain and a MAPKKK domain in the central region, and a WD40 domain in the C-terminus [17, 18]. Both *α*S and LRRK2 (PARK8) are involved in various pathologies such as impairment of cytoskeleton dynamics and dysregulation of the protein degradation system [19]. Mitochondria may also be a common target of *α*S and LRRK2, since immunoreactivities of LRRK2 are associated with mitochondria and with other membranous and vesicular intracellular structures, including lysosomes, endosomes, and transport vesicles [20]. Furthermore, it has been shown that LRRK2 regulates mitochondrial dynamics by increasing mitochondrial DLP1 (dynamin-like kinase 1) through a direct interaction with DLP1 [21].

In most autopsies, brains with the LRRK2 G2019S mutation have Lewy body pathologies, as opposed to LRRK2 non-G2019S carriers, but there is marked variability in pathological findings, even among carriers with identical mutations, indicating that additional mechanisms may be critical [22]. Thus, *α*S and LRRK2 may cooperate with each other to produce diverse pathologies, including mitochondrial dysfunction, but the relationship between these two proteins might be more complicated. 

## 4. Mitochondrial Dysfunction in Axonal Swellings in Mice Models of **α**-Synucleinopathies

Axonopathy is critical in the early stage of pathogenesis of neurodegenerative diseases, including *α*-synucleinopathies, and it has been shown that axonal swellings, including globules and spheroids, are characteristic features of axonopathies [23]. Since transgenic (tg) mice expressing *α*S or DLB-linked P123H *β*S are characterized by formation of similar axonal globules [24], they were histologically analyzed to compare the pathologies, including mitochondrial function, caused by accumulation of the two different types of synucleins [8].

A recent study suggests that dysfunction of the autophagy-lysosome pathway may be one of the contributors to axonal swellings. Failure to degrade subcellular materials or organelles at distal axons and/or nerve terminals or failure to export these materials by axonal transport has been shown to produce swollen nerve terminals. Such a mechanism might be involved in formation of both *α*S- and P123H *β*S-globules. In our recent study, *α*S-globules in brains of *α*S tg mice were characterized by autophagosome-like membranous elements and were immunopositive for various minor gangliosides, which is reminiscent of some types of lysosomal storage disease [8]. Consistent with this, lysosomal activity, as assessed by the activities of cathepsins B and D, was significantly decreased in brain extracts of *α*S tg mice compared with those from non-tg littermates [8]. Similar lysosomal dysfunctions have been observed for P123H *β*S-globules in brains of P123H *β*S tg mice [24]. These results suggest that downregulation of the lysosome degradation pathway may be a common mechanism leading to globule formation in *α*S and P123H *β*S tg mice.

**Figure 2:**
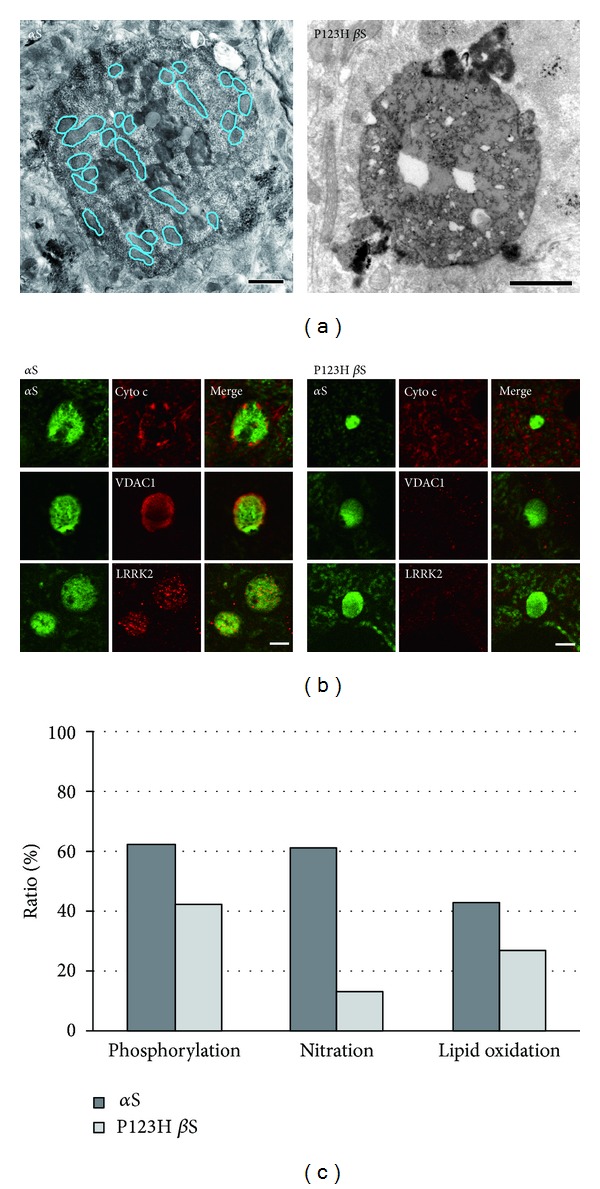
Axonal swellings in two types of synucleinopathy model mice. Immunoelectron microscopic analysis was performed using anti-*α*S. *α*S-immunopositive globules (a) were characterized by lysosomal pathologies such as myelinosomes (in *α*S mice) and lipid droplets (in P123H *β*S mice). Accumulation of mitochondria was occasionally observed only in *α*S mice (a: blue). Because P123H *β*S-immunopositive globules in brains of P123H *β*S tg mice were immunopositive for *α*S (~100%), double immunofluorescence analyses of *α*S tg mice (b: nine left panels) and P123H *β*S tg mice (b: nine right panels) were performed using *α*S as a globule identification. In *α*S tg mice, cytochrome c (b: upper panels) showed punctate patterns, while VDAC1 (b: middle panels) was located diffusely. In P123H *β*S tg mice, cytochrome c and VDAC1 were all immunonegative. Note that *α*S-globules were immunopositive for LRRK2 (b: lower panels), whereas P123H *β*S globules were negative for LRRK2. Quantification of data for phosphorylation of *α*S, nitration of *α*S, and lipid oxidation (immunoreactivity for 4-hydroxy-2-nonenal) in the *α*S-globules of both of synucleinopathy model mice (c). Scale bar = 1 *μ*m for (a); 5 *µ*m for (b). Please see [8] Mol. Brain for detailed information; (reprinted from Mol. Brain, Sekigawa et al., 5 : 34 with permission).

In contrast to the lysosomal pathology, mitochondria accumulated specifically in *α*S-globules [8] (Figure 2(a)). Some *α*S-globules displayed clustering of mitochondria, while others had swollen mitochondria in the peripheral regions. Immunoreactivities of mitochondrial markers such as VDAC1 and cytochrome c were also found in *α*S-globules (Figure 2(b)). These results suggest that mitochondria clustering might become hyperactive in response to lysosomal dysfunction. Consistent with these findings, *α*S-globules were associated with oxidative stress, as assessed by staining of 4-HNE and nitrated *α*S. Conversely, no evidence of mitochondria was obtained in P123H *β*S-globules; hence oxidative stress (assessed by 4-HNE staining) was less than that in *α*S-globules [8] (Figure 2(c)).

Notably, LRRK2 was located in *α*S-globules and may be actively involved in the axonal pathology. The specific accumulation of LRRK2 in *α*S-globules may corroborate the idea that LRRK2 cooperates with *α*S in the axonal pathology. Indeed, it has been shown that LRRK2 is crucial for regulation of neurite formation and length [25]. Knockdown of LRRK2 led to long and highly branched neuritic processes, whereas constructs with increased kinase activity exhibited short simple processes in neuronal cultures (or transduced nigrostriatal models) [25]. More recently, LRRK2 R1441G BAC tg mice were shown to have various characteristic axonal pathologies, including large tyrosine hydroxylase-positive spheroid-like structures, dystrophic neuritis, and enlarged axonal endings [26].

## 5. Summary and Perspective: A Heterogeneity Problem?

As far as we are aware, there has been only one previous description of abnormal mitochondria in the axonal pathology in tg mice expressing prion promoter-driven *α*S [27]. Thus, the results of our studies in tg mice provide novel information showing that *α*S-globules derived from *α*S tg mice and P123H *β*S-globules derived from P123H *β*S tg mice have similar (e.g., lysosomal pathology) but distinct characteristics (e.g., mitochondrial alteration and LRRK2 accumulation). 

P123H *β*S may represent a rare cause of familial DLB [28] but might reflect a process in which wild type *β*S becomes pathogenic in aging and may be involved in axonal pathology (e.g., formation of axonal globules) in sporadic cases of *α*-synucleinopathies [29, 30]. In support of this view, neurite accumulation of *β*S has been demonstrated in various synucleinopathies, including PD, DLB, and neurodegeneration with brain iron accumulation, type I [31, 32]. Furthermore, the gracile axonal dystrophy (gad) mouse, which has a naturally deleted ubiquitin carboxy-terminal hydrolase-1 (UCH-L1), is characterized by formation of spheroid bodies in nerve terminals, which are negative for *α*S but positive for *β*S or *γ*S [33]. Based on the results of our transgenic studies, one may speculate that the swellings with *β*S, but not those with *α*S, might be generally free from mitochondrial pathology and LRRK2 accumulation. Taken together, these results suggest that the synuclein family of peptides might contribute to formation of axonal pathologies through diverse effects on mitochondrial and LRRK2 pathologies.

The diversity of pathology, including mitochondrial pathology, is a hallmark of the pathogeneses of neurodegenerative diseases. However, little attention has been paid to this key issue. We believe that this could be important not only from a mechanistic perspective but also therapeutically. It has generally been believed that mitochondria represent a potential target for development of disease-modifying therapies. Following this logic, clinical trials of antioxidants, including Vitamin E (*α*-tocopherol), have been controversial for treatment of neurodegenerative disorders [34, 35]. Based on our observations, this could be at least in part due to the heterogeneity of mitochondrial pathologies caused by the synuclein family of peptides. If this view is correct, there is a need to establish a new strategy to overcome such diversity of neuropathologies, including mitochondrial dysfunction, in the treatment of neurodegenerative diseases.
